# Select Endocrine Disorders and Exosomes in Early PDAC Diagnosis

**DOI:** 10.3390/ijms252212159

**Published:** 2024-11-13

**Authors:** Barbara Wlodarczyk, Lukasz Durko, Konrad Walczak, Renata Talar-Wojnarowska, Ewa Malecka-Wojciesko

**Affiliations:** 1 Department of Digestive Tract Diseases, Medical University of Lodz, 90-153 Lodz, Poland; 2 Department of Internal Diseases and Nephrodiabetology, Medical University of Lodz, 90-549 Lodz, Poland

**Keywords:** pancreatic ductal adenocarcinoma, new-onset diabetes, exosomes, pancreatic cancer biomarkers

## Abstract

Disturbances in carbohydrate metabolism are suggested to be the early symptoms of pancreatic ductal adenocarcinoma (PDAC). The accumulated data suggests that endocrine function-related biomarkers may represent a breakthrough in the early detection of PDAC. Factors which may predispose one to the development of PDAC are insulin resistance and hyperinsulinemia. Elevated insulin levels induce the onset of carcinogenesis by altering the differentiation and function of islet cells through stimulating growth factors, including insulin-like growth factors (IGFs). Impaired β cell function, along with the impact of PDAC-released factors (e.g., adrenomedullin (ADM), IGF-1, and macrophage inhibitory factor (MIF) on pancreatic islets, may contribute to the induction of diabetes associated with PDAC. Recently, exosomes have attracted worldwide attention due to their role in varied features of cell function, particularly in cancer progression. Exosomes comprise of small extracellular vesicles produced by almost all cells. These vesicles contain a vast array of biomolecules, including proteins and microRNAs. Exosomes participate in cancer growth and promote angiogenesis. They promote tumorigenesis and metastasis, and are associated with the acquisition of cancer cells resistant to chemotherapy. Data have been accumulating recently on the role of exosomes in the rapid recognition, prognosis and potential therapy of pancreatic cancer.

## 1. Introduction

Pancreatic ductal adenocarcinoma (PDAC) is the fourth leading cause of cancer deaths in men and fifth leading cause among women [1]. The vast majority of pancreatic neoplasms (95%) originate from the exocrine cells. Among exocrine tumors, 85–90% are PDAC, usually being located in the head of the pancreas, while less than 10% of the pancreatic focal lesions are cystic neoplasms [2]. PDAC is detected with a slight predominance in men over 60 years of age [3]. It has been shown that smoking, being overweight, obesity, long-term diabetes mellitus (DM) type 2 and chronic pancreatitis (CP) increase the risk of this cancer [4].

PDAC develops from the progression of various precursor lesions in the pancreatic duct epithelium, especially pancreatic intraepithelial neoplasia (PanIN), but also intraductal papillary mucinous neoplasms (IPMNs), mucinous cystic neoplasms (MCNs), and possibly atypical flat lesions (AFLs) [5]. PanIN is characterized as a microscopic flat or papillary noninvasive epithelial lesion which develops in small pancreatic ducts measuring less than 5 mm in diameter. These lesions consist of cuboidal or columnar cells containing varying amounts of mucin, which aids in distinguishing them from normal ductal epithelium, made up of cuboidal or low columnar cells with amphophilic cytoplasm and lacking any evidence of mucinous cytoplasm [6]. Based on the level of architectural and cytological atypia in pancreatic ducts, PanINs are classified into four grades; the lowest grades are PanIN 1A and PanIN 1B, followed by PanIN 2, which represents intermediate-grade PanINs, and PanIN 3, the high-grade category. PanIN 3 is considered the highest grade and is also known as carcinoma in situ [7].

The majority of pancreatic cancers are sporadic. Only 10% of PDACs are associated with hereditary syndromes, like hereditary pancreatitis, familial PDAC, Peutz–Jeghers syndrome, Lynch syndrome (HNPCC), familial adenomatous polyposis (FAP), hereditary breast and ovarian cancer (HBOC), familial-atypical multiple mole melanoma (FAMMM), Fanconi anemia, Hippel–Lindau disease, Li–Fraumeni syndrome, and an ataxia telangiectasia [8,9].

About 80–85% of patients with PDAC are diagnosed at a stage of locally advanced or metastatic disease, making radical surgical treatment unfeasible [10]. Diagnosis is directly related to symptoms occurrence, such as abdominal pain, weight loss, or jaundice. At the early stage, PDAC does not cause any worrisome symptoms and may only be detected accidentally, in imaging diagnostics performed for other indications. Difficulties in detecting pancreatic cancer in early stages delays the initiation of therapy. As a result, PDAC is one of the cancers with the worst prognosis, with a 5 year survival rate of about 12% and a median survival rate of about 6 months, and its incidence is almost equal to the mortality rate. Another reason for PDAC lethality, is its high metastatic potential and resistance to chemotherapy [2,3].

The association of diabetes and PDAC is the most widely observed and studied one, but also in other cancers of the digestive tract, diabetes can also develop. Recently, lots of studies have shown that certain cancers expand more frequently in patients with diabetes [11]. Type 2 DM is connected with a higher risk of many cancer, including non-Hodgkin’s lymphoma, colorectal cancer, liver cancer, and bladder cancer [12,13,14,15]. The prevalence of diabetes in PDAC ranges from 4% to as much as 65%. Nearly 85% of pancreatic cancer patients have hyperglycemia or new onset diabetes (NOD) (75%) [16,17,18,19]. Recent studies reported that 4–20% individuals with PDAC had a previous history of diabetes. The pathophysiological links between glycemic regulation and the susceptibility to developing PDAC are intricate. Prolonged DM type 2 is linked to approximately a twofold higher risk of pancreatic cancer development. In this scenario, there is a systemic pro-inflammatory environment which could facilitate neoplastic changes in the pancreas [20]. NOD induced by PDAC is associated with pro-inflammatory alterations, insulin resistance, and dysfunction in β cells, leading to a disruption in glucose homeostasis [21]. In NOD, the β cells’ capacity to display typical insulin secretory patterns is disturbed. The islet blood flow features physiological fenestrations which make it “leaky”, a characteristic which could be exacerbated by the aforementioned disruptions, consequently worsening intra-pancreatic insulin stasis [22]. A study by Zhang et al. described a mouse model designed to investigate the role of insulin receptor signaling in the initiation of a high-fat diet (HFD), which is known to produce sustained hyperinsulinemia and accelerate PanIN and PDAC development. All mice were fed a HFD to induce long-term hyperinsulinemia and accelerate PanIN and PDAC development. At 4 weeks of age, tamoxifen was injected to induce mutant KRAS^G12D^ and nuclear green fluorescent protein (GFP) expression and eliminate floxed insulin receptor (Insr) alleles, particularly in acinar cells. The authors studied mice with a full Insr gene dosage, mice with partially reduced Insr levels, or knockout and mice without any Insr in KRAS^G12D^-expressing acinar cells. It was that hyperinsulinemia-induced promotion of PDAC initiation is mediated by direct Insr signaling in pancreatic acinar cells. Research revealed that the mice on a HFD exhibited significant PanIN development and tumor progression when Insr was specifically absent in the KRAS^G12D^-expressing acinar cells. These findings indicate that hyperinsulinemia directly facilitates the initiation of pancreatic cancer through Insr in acinar cells, likely via a mechanism which involves increased production of digestive enzymes and subsequent pancreatic inflammation. It was indicated that acinar-specific Insr, deletion does not significantly affect glucose regulation in mice. This model allowed for the examination of the role of acinar cell Insr in the context of hyperinsulinemia and normal blood sugar levels [23]. The use of Insr in detecting pancreatic cancer is discussed in animals, but it can also be used in humans. In a study by Heckl et al. study, a cohort of pancreatic cancer patients showed that insulin can induce the expression of programmed death-ligand 1 (PD-L1), a crucial molecule in immune checkpoint regulation. This effect of insulin is significantly dependent on Insr [24].

A widely proven compound that inhibits the development of diabetes and pancreatic cancer is metformin. The pathways and targets of metformin are further examined, highlighting both its direct actions on transformed pancreatic epithelial cells and its indirect, systemic influences on extra-pancreatic tissues [25].

The significantly elevated insulin levels overly stimulate IGF-1 and both the Insr and the IGF-1R, resulting in increased survival of pre-malignant cells, heightened cancer cell proliferation, and resistance to apoptosis. Disturbances in blood flow through the islets, microthrombosis, and perivascular fibrosis in PDAC likely combine to suppress the ability of β cells to exhibit normal insulin secretion dynamics in NOD [22].

The role of proteins in circulating exosomes as biological markers for pancreatic cancers has been investigated in multiple studies [23,26]. PDAC release exosomes which may carry proteins, like e.g.,: adrenomedullin (ADM) and macrophage inhibitory factor (MIF), which cause β cell dysfunction and decreased insulin secretion [26,27]. MIF is highly expressed in exosomes derived from pancreatic cancer. Patients with stage I PDAC who later developed liver metastasis showed a significant increase in MIF compared with those without tumor progression, indicating the important role of exosomal MIF in liver metastasis and its potential as a prognostic marker for predicting liver metastasis [26,28]. Javeed et al. characterized exosomes from the conditioned media of PDAC cell lines and the portal and peripheral venous blood of PDAC patients. Exosomes were identified as the primary extracellular vesicles released by PDAC into culture media and human plasma. The PDAC-derived exosomes contained ADM and CA19-9, entered β cells through caveolin-mediated endocytosis or macropinocytosis, and suppressed insulin secretion. The ADM present in PDAC-derived exosomes interacted with its receptor on β cells. ADM receptor suppression abolished the inhibitory effect of exosomes on insulin secretion. In addition, β cells exposed to ADM or PDAC-derived exosomes exhibited increased expression of endoplasmic reticulum (ER) stress genes and elevated levels of reactive oxygen and nitrogen species. A modest increase in insulin mRNA was observed in response to ADM. ER stress is a physiological response of β cells to increased insulin production, whereas an efficient unfolded protein response (UPR) safely resolves ER stress. The question of whether the paradoxical inhibition of insulin secretion by ADM, despite the increase in cyclic adenosine monophosphate (cAMP), could be due to independent, ADM-induced unresolved ER stress resulting from an unsuccessful UPR remains [27].

Therefore, the aim of this review is to assess the current evidence concerning the role of NOD in the early diagnosis of PDAC. Additionally, we provide literature data suggesting the potential role of exosomes as biomarkers for both PDAC and PDAC-associated diabetes.

## 2. The Role of the New-Onset Diabetes in Early PDAC Detection

**Figure 1:**
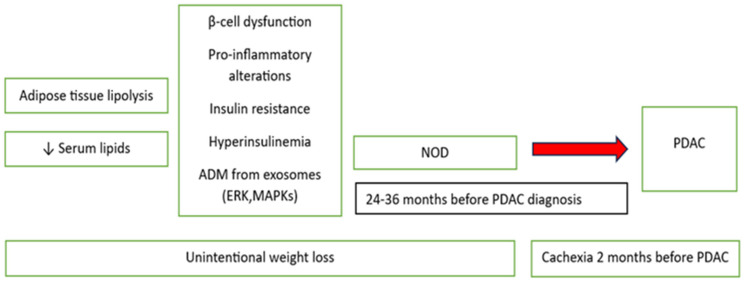
The hypothesis on the role of NOD in PDAC development. Adrenomedullin (ADM), new-onset diabetes (NOD), pancreatic ductal adenocarcinoma (PDAC), endoplasmic reticulum kinase (ERK), and mitogen-activated protein kinases (MAPKs), ↓ means lower level/decrease.

NOD, which is diabetes-identified 24–36 months before PDAC diagnosis, as well as hyperinsulinemia with unintentional weight loss, may be the first symptoms of cancer [29,30,31,32]. An increase in the blood glucose concentration, reduction in body weight, and decreased serum lipids levels were identified in the 6–18 months preceding PDAC diagnosis [33]. Early weight loss in PDAC begins many months prior to the onset of cachexia. It is paradoxically associated with the development of NOD and likely due to adipose but not muscle tissue loss, with the latter being a hallmark of cachexia [34]. Sagar et al. proposed that NOD and the associated weight loss in PDAC are paraneoplastic phenomena caused by tumor-secreted products derived from exosomes. Adipose tissue lipolysis might explain early onset weight loss in PDAC. Moreover proteins from exosomes, like ADM, interact with their receptor on the adipocytes, activate p38 and extracellular signal-regulated kinases (ERK) 1 and 2, and mitogen-activated protein kinases (MAPKs) and promote lipolysis [35]. In many cancers, the dysmetabolic state, and diabetes may occur, but their particularly high prevalence in PDAC compared with other cancers suggests a unique relationship [36]. In addition, the onset of diabetes in PDAC occurs 2–3 years prior to the diagnosis of cancer, whereas cachexia-associated symptoms occur 2 months prior to cancer diagnosis on average (Figure 1) [19].

The integrative metabolic function of myokines and adipokines has recently been emphasized by the demonstration that interleukin 6 (IL-6), derived from either muscle or adipose tissue in the context of obesity, facilitates communication between intestinal cells and pancreatic islets [37,38]. It adjusts insulin secretion for different metabolic states (e.g., malnutrition, obesity, or DM type 2) [39,40]. IL-6 promotes glucagon-like peptide-1 (GLP-1) production and secretion from intestinal L cells and pancreatic α cells, leading to improved β cell insulin secretion and glucose tolerance. These findings are significant for cachexia, as the sustained high levels of IL-6 in advanced cancer patients may interfere with the normal regulatory functions of IL-6 in managing glucose tolerance, fatty acid (FA) and amino acid metabolism, insulin action, and ultimately body mass [36]. IL-6 possesses anti-inflammatory characteristics and can collaborate with insulin signaling to enhance activities in muscles as well as induce lipolysis in adipose tissue [41]. Conversely, sustained elevated levels of IL-6, induced by the introduction of IL-6, lead to insulin resistance and decreased body mass [42]. Newly diagnosed diabetes resulting from PDAC is linked to pro-inflammatory changes, insulin resistance, and disturbances in β cell function, which ultimately result in the loss of glucose homeostasis. Adipocytes and resident macrophages secrete numerous detrimental pro-inflammatory factors, while increased lipolysis can elevate the production of acetyl-CoA in the liver, leading to hepatic insulin resistance and excessive glucose release from the liver [21].

Some studies have shown that the onset of diabetes does not depend on the stage of the tumor, its size, or its location [16,40]. It has been suggested that endocrine disorders occur in the early stage of pancreatic cancer [40,43]. In Pannala et al.’s study, 512 newly-diagnosed PDAC cases and 933 controls were prospectively recruited. DM was more prevalent (47% versus 7%, *p* < 0.001) among the PDAC cases compared with the controls. Similarly, NOD (<2 year duration) was predominant among PDAC cases compared with the controls (74% versus 52%, *p* = 0.002). The prevalence of DM was not significantly associated with the tumor’s location (head versus body or tail: 72% versus 66%, *p* = 0.2) or tumor stage (stage I or II (47%) versus stage III or IV (43%), *p* = 0.2). In this study, the median survival was similar in PDAC with and without DM. However, the effectiveness of using hyperglycemia as a screening tool to identify individuals with a high likelihood of asymptomatic PDAC will significantly rely on the ability to distinguish PDAC-associated diabetes from the more prevalent type 2 DM. Some researchers proposed that PDAC should be considered in patients with NOD who are lean, healthy, have no family history of diabetes, meaning patients with NOD who lack the risk factors for type 2 DM [16,40,43].

Insulin resistance may be caused by a hormonal factors, like islet amyloid peptide (IAAP). Several islet peptides normally produced by the endocrine pancreas, such as glucagon, somatostatin, and IAPPs, have a diabetogenic effect. IAPP inhibits glucose uptake and glycogen synthesis in skeletal muscle both in vitro and in vivo, as well as in the liver in vivo. In animals, IAPP administration can lead to impaired glucose tolerance. IAPP is a 37 amino acid polypeptide, produced in islet β cells and is a major component of the pancreatic amyloid found in insulinomas and in the pancreases of 90% of diabetic patients. IAPP is secreted with insulin, but the secretion of these two hormones is independently controlled under certain conditions, such as when islet cells are transformed [44]. The tissue-specific expression of IAPP and insulin is regulated differently, and the co-expression of IAPP with hormones other than insulin may serve as a marker for pluripotent transformed rat islet cells. The IAAP circulating concentration is elevated in cases of PDAC with accompanying diabetes [44,45]. In Permert et al.’s study, the concentrations of IAPP in the serum were elevated in PDAC patients compared with healthy individuals (22.3 ± 13.6 versus 8.0 ± 5.0 pmol/L, *p* < 0.001). In PDAC, the IAPP concentrations were 25.0 ± 8.7 pmol/L in seven patients with diabetes requiring insulin, 31.4 ± 12.6 pmol/L in 11 patients with diabetes not requiring insulin, and 12.2 ± 2.4 pmol/L in 9 patients with normal glucose tolerance. They concluded that elevated plasma IAPP levels are observed in pancreatic cancer patients with diabetes. The overproduction of IAPP, which can induce insulin resistance, may play a role in the development of diabetes in these individuals [44]. On the other hand, IAPP declines with diabetes progression. It has been suggested that IAPP acts as a tumor suppressor in p53-deficient cancers. Considering the decrease in IAPP levels during diabetes advancement, it was explored the effects of IAPP in PDAC were explored to assess whether the loss of IAPP in diabetes could elevate the risk of pancreatic cancer. In Taylor et al.’s study, PANC-1, MIA PaCa-2, and H1299 cell cultures were exposed to rodent-derived IAPP, as well as the IAPP analogs pramlintide and davalintide, and assessed for changes in proliferation, apoptosis, and glycolysis. A mouse model lacking IAPP was created for survival analysis in PDAC. IAPP did not affect glycolysis in MIA PaCa-2 cells and did not influence cell apoptosis, proliferation, or glycolysis in PANC-1 cells or H1299 cells, which were previously identified as responsive to IAPP. Deletion of IAPP in mice did not impact their survival time in the presence of a lethal tumor burden. It was concluded that IAPP does not act as a tumor suppressor, indicating that the absence of IAPP signaling is unlikely to raise the risk of pancreatic cancer in individuals with diabetes [46].

NOD, significant weight loss with normal blood glucose levels, and rapid development of hyperglycemia before the onset of diabetes are potential markers of pancreatic cancer. In a case control study among subjects with NOD, adjusted odds ratios (aORs) for PDAC as a function of both weight change and blood glucose before the onset of diabetes were calculated. Weight loss ranging from 10.0% to 14.9% at the onset of diabetes was linked to an aOR for pancreatic cancer of 3.58 (95% CI, 2.31–5.54), while a loss of ≥15.0% was associated with an aOR of 4.56 (95% CI, 2.82–7.36) compared with maintaining a stable weight. Blood glucose levels of ≤5.1 mmol/L or 5.2–5.6 mmol/L before the onset of diabetes were correlated with an increased risk of being diagnosed with pancreatic cancer, with aORs of 2.42 (95% CI, 1.60–3.66) and 2.20 (95% CI, 1.45–3.35), respectively, when compared with blood glucose levels ≥6.3 mmol/L more than 2–3 years before cancer detection [47]. In our earlier study, the glucose and insulin levels were analyzed with the oral glucose tolerance test (OGTT) in three groups: patients with CP, patients with PDAC, and in healthy subjects. Moreover, in all subjects, we assessed insulin sensitivity with the homeostatic model assessment for insulin resistance (HOMA). Diabetes was diagnosed in 44.4% of patients with CP and 27.8% of patients with PDAC. When evaluating the mean values of insulin fasting, after 60 and 120 min of the test, we showed that the level was the highest in the control group (10.2 ± 7.9 IU/mL fasting, 33.1 ± 21.4 IU/mL at 60 min, and 34.1 ± 44.7 IU/mL at 120 min). In the CP and PDAC groups, at the beginning of the test, these values were similar (3.3 ± 1.3 IU/mL in the CP group and 3.6 ± 1.8 IU/mL in the PDAC group). After 60 min, the level of insulin in the CP patients was 17.5 ± 6.9 IU/mL, while that in the PDAC group was 22.3 ± 17.4 IU/mL. At the 120th minute of the study, a significant increase in the level of insulin was observed in the PDAC group (31.9 ± 19.3 IU/mL) compared with the CP group, where the result was 16.1 ± 7.2 (*p* < 0.05). The average HOMA value in the CP group was low, being at the level of 0.80 ± 0.4, which was similar to that of the PDAC group (0.83 ± 0.4), showing normal insulin sensitivity in those patients. In the study, the OGTT test allowed for the detection of endocrine disorders in patients with CP and PDAC, despite the absence of clinical symptoms of diabetes. Overall, in the PDAC group, hyperinsulinemia was found in 50% of cases and in 59% CP patients. In addition, in the CP patients, reduced insulin secretion was observed only in patients with impaired fasting glucose (IFG) and impaired glucose tolerance (IGT). We found that in the CP group, most patients did not suffer from obesity, lipid disorders, hypertension, or other features of the metabolic syndrome, which is characteristic of the pathomechanism of insulin resistance accompanied by a chronic inflammatory process [48].

Sharma et al. compared the glycemic profiles of patients with PDAC (cases) to age- and gender-matched individuals without cancer (controls). The authors noted that the majority of tumors were not spherical (<10%) and typically had three distinct dimensions. Consequently, in post-surgery samples, they computed the tumor volume as that of a scalene ellipsoid (4/3Π r1 × r2 × r3), considering all three tumor dimensions. Across each ellipsoid volume category, there was a broad variation in the largest tumor diameter. The authors analyzed FBG profiles collected over 60 months prior to PDAC diagnosis in patients with PDAC (*n* = 219) (cohort A), FBG profiles of subjects with resected PDAC (*n* = 526) stratified by tumor volume and grade (cohort B), and FBG profiles of patients with resected PDAC and long-term FBG (*n* = 103) (cohort C). The primary endpoint was to estimate the time to invasive PDAC based on hyperglycemia, which was defined as being significantly higher (*p* < 0.05). The mean FBG in cohort A did not significantly differ between the cases and the control group 36 months before PDAC diagnosis. Hyperglycemia was observed 36–30 months before PDAC in all cases. In cohort B, the mean FBG did not significantly differ in the control group compared with the cases with PDAC below 1.0 cc. The smallest tumor volume associated with hyperglycemia was 1.1–2.0 cc, where FBG levels increased with the tumor volume. The FBG levels varied depending on the tumor grade; the well- or moderately differentiated tumors (5.8 cc) exhibited similar FBG levels to smaller, poorly differentiated tumors (1.5 cc) (*p* < 0.001). In cohort C, the duration of hyperglycemia in cases of PDAC with large, moderate, or small volumes was 36–24, 24–12, and 12–0 months, respectively. Furthermore, the authors confirmed that PDAC resection lowered the level of hyperglycemia, regardless of the tumor’s location. In summary, in a clinical control study of patients with PDAC, FBG levels were correlated with the time to PDAC diagnosis, tumor volume, and grade. All of these findings strongly suggest hyperglycemia being a biomarker of early invasive PDAC, mostly for NOD [49].

In another study, the mean interval between the onset of diabetes and the diagnosis of pancreatic cancer was shorter, being 10 months (ranging from 5 to 29 months). It has also been demonstrated that when a patient’s fasting glucose levels exceed the diabetes threshold (≥126 mg/dL), nearly half of the patients have a potentially resectable tumor. These findings indicate that the resectability of pancreatic cancer can be significantly enhanced if it is identified at least six months before its clinical diagnosis, suggesting that diabetes may manifest at a resectable stage of the disease. Unfortunately, there is currently no serological marker available which can differentiate diabetes associated with pancreatic cancer from type 2 diabetes [50].

In PDAC patients, differentiating type 2 diabetes from cancer-associated diabetes (type 3c diabetes) will be essential for limiting the screening target group, since DM type 2 is widely spread in the population. The authors hypothesized that individuals with PDAC may first exhibit IGT, characterized by the initial signs of insulin resistance, before the diabetes develops. It is proposed that the available database of the high PDAC risk population in combination with NOD be expanded. To distinguish cases of early-stage cancer from the general population, a highly accurate test with a specificity greater than 99% would be required. A more practical approach would involve starting with a subgroup of the population with a high frequency of PDAC occurrence, combined with new markers to narrow down the risk subgroup. This could help identify a group with significantly elevated PDAC risk, allowing for the implementation of a screening strategy based on endoscopy and imaging [51]. Sharma et al. aimed to develop and validate a model to determine the risk of pancreatic cancer in individuals with NOD. In the examined cohort, the clinical model called Enriching New-Onset Diabetes for Pancreatic Cancer (END-PAC), identified patients who developed pancreatic cancer within 3 years after the onset of diabetes, with an area under the ROC value of 0.87, a cutoff score of ≥3, had a sensitivity of 80%, and specificity of 80%. The model was designed in such a way that three points were awarded for symptoms: age (≥50 years of age), changes in glucose level (100–109 mg/dL and >160 mg/dL), and changes in weight loss of ≥2.5 kg. In the study, a score which stratified subjects into a high-, intermediate-, or low-risk groups for pancreatic cancer at the time they first met the glycemic criteria for diabetes was validated. The incidence of pancreatic cancer within 3 years in individuals with glycemia defined as NOD was ~1% and increased to 3.6% in individuals with an END-PAC score ≥3 (END-PAC cohort), requiring clinical evaluation. A negative END-PAC result (≤0) had a notably high negative predictive value for pancreatic cancer. Patients with these results should be managed as type 2 diabetes patients. The remaining 25% of patients with PDAC and NOD had an END-PAC score of one or two; the 3 year incidence of pancreatic cancer in this group was 0.5%. Biomarkers in this group may help enrich this cohort for pancreatic cancer [52].

## 3. Characteristic of Diabetes in CP

The true prevalence of pancreatogenic diabetes represented 1–9% of all DM cases. The vast majority of them were associated with CP (75%), and 9% were associated with PDAC (second cause) [53]. The development of endocrine disorders in pancreatitis has different mechanisms than PDAC. In patients with CP, the dynamics of the carbohydrate metabolism disorders depend on the etiology and duration of the disease [54]. In alcoholic CP, diabetes usually appears at an advanced stage with fibrosis and the presence of calcifications in 10–35% of patients after about 8–10 years of the disease’s duration [46]. It was assumed, that endocrine disorders in CP result from a decrease in insulin secretion caused by progressive fibrosis of the organ and damage to and the loss of both, α and β cells in pancreatic islets [55]. The dysfunction of pancreatic α cells is manifests with the insufficient response of glucagon to hypoglycemia and its incomplete suppression after oral glucose administration, which is similar to that of patients with type 2 DM. In a study by Mumme et al., based on the hypoglycemic clamp and OGTT, 10 patients with diabetes secondary to CP were compared to 13 patients with type 2 DM, with 10 healthy individuals as a the control group. The glucose levels during the OGTT were significantly higher in the patients with diabetes and CP compared with the control subjects (*p* < 0.0001). Insulin and C-peptide levels were decreased, and the glucose-induced suppression of glucagon was impaired in both diabetes groups (all *p* < 0.0001 versus. control subjects). During hypoglycemia, the glucagon concentrations were lower in the patients with CP and type 2 diabetes (*p* < 0.05). The increase in glucagon during the clamp was inversely correlated with glucose-induced glucagon suppression and positively correlated with β cell function. In conclusions, α cells responses to oral glucose ingestion and to hypoglycemia were disturbed in the patients with diabetes and CP and in the patients with type 2 DM [56].

It has been proven that, diabetes in the course of CP is accompanied by concomitant insulin, glucagon, and pancreatic polypeptide (PP) deficiency [55,56]. Nevertheless, β cell dysfunction may occur early in CP long before islet destruction [57]. The potential mechanism involves activated stellate cells’ products or toxic factors, including inflammatory cytokines produced in the pancreas, crossing into the islets and disrupting the functioning of β cells. The mechanism of β cell failure in the early stages of CP development is not associated with massive destruction of islets but rather with their progressive dysfunction [55,56,58]. A potential mechanism of this phenomenon involves the impairment of β cell function by inflammatory cytokines such as IL-1β, and IL-6 activated by lipocalin-2 (LCN2) within stellate cells [59]. It is different than in advanced CP, which showed a reduction in and destruction of islet β cell mass in histological examination and reduced glucose-stimulated insulin release. In Mitnala et al.’s study, expression of the pancreatic and duodenal homeobox gene (*PDX-1*) was assessed to evaluate the function of α cells in islets from CP patients. Islets were isolated from the pancreases of 40 patients undergoing surgical procedures, classified into a control group and patients with mild and advanced CP, and evaluated for their efficiency, size, and glucose-stimulated insulin secretion. Concurrently, the expression of genes encoding *PDX-1*, insulin, and glucagon was monitored. Compared with the control group, the islet efficiency did not significantly differ in the patients with mild CP but was significantly reduced in the patients with advanced CP. Although a marginal decrease in islet size was observed in the patients with mild CP, it did not significantly differ from that observed in the control group. In patients with advanced CP, a 58% reduction in size was observed, accompanied by a significant decrease in α cell mass. The expression of insulin and *PDX-1* genes (but not glucagon) was significantly diminished in the patients with advanced CP. The islets harvested from patients with advanced CP maintained 53% of their glucose-stimulated insulin secretion function when compared with the control group [60].

In the pathogenesis of the development of endocrine insufficiency in patients with CP, abnormal functioning of incretin hormones can be found. Glucagon-like peptide-1(7–36)amide and GIP, produced by endocrine cells of the intestinal mucosa, stimulate insulin release, playing a role in regulating postprandial nutrient homeostasis. Both incretins are released in response to glucose, lipids, or a mixed meal in amounts proportional to the meal size and stimulate insulin secretion in the presence of elevated blood glucose levels [61]. These two incretins are glucose-dependent stimulators of pancreatic β cell function, exhibiting a range of secondary extra-pancreatic actions which support effective control of blood glucose homeostasis in response to the appearance of nutrients in the duodenum broken down by pancreatic enzymes. GLP-1 and GIP possess several characteristics which extend beyond their synthesis in the intestines. Both hormones send signals through coupling with G protein-coupled receptors found on islet β cells, stimulating insulin release only when supraphysiological glucose levels are present. Additionally, their insulinotropic effects are inactivated by the enzyme dipeptidyl peptidase-4 (DPP-4) [62]. Defects in nutrient regulation of insulin secretion, resulting from the lack of pancreatic enzymes and resistance to the cellular actions of insulin, are key to the development of non-insulin-dependent diabetes [58]. The entero-insular axis, consisting of incretin hormones and neural pathways, plays a crucial role in regulating insulin secretion. The term “incretin” refers to a humoral factor released from the intestinal mucosa which stimulates insulin secretion and has provided evidence that insulin can be released due to stimulation of the vagus nerve. This confirms the involvement of the nervous system as a mechanism controlling gastrointestinal function. It has been found that many gastrointestinal peptides (also with potential insulin-releasing effects, such as incretins) are present in the central nervous system and the gut, including cholecystokinin (CCK), enkephalin, gastrin, neurotensin, somatostatin, substance P, thyrotropin-releasing hormone (TRH), and vasoactive intestinal peptide (VIP). These peptides can act as neurotransmitters. In addition to the known adrenergic and cholinergic systems, the peptidergic nervous system, including the vagal and cholinergic nerves, must be considered [63,64]. The gastrointestinal nervous system may participate in the gut-islet axis in two ways: by releasing incretins into the blood, and second, by directly stimulating pancreatic islets through nerve fibers [64]. GIP and GLP-1 act on β cells to promote glucose-dependent insulin release by activating protein kinase A (PKA) and the related pathways [65]. These enzymatic systems are also triggered by specific receptors for hormonal and neural components of the entero-insular axis, resulting in the activation of PKA and PKC. This activation promotes protein phosphorylation and enhances the sensitivity of the secretory process to the secretory action of Ca^2+^ [58].

## 4. Pancreatogenic Diabetes in the Course of PDAC

Currently, two hypotheses have been proposed to explain the phenomenon of frequent coexistence of PDAC and diabetes. According to the first hypothesis, DM is a risk factor for the development of pancreatic cancer, while the second assumes that it is a consequence of PDAC development [16,30,32,49]. While long-standing type 2 DM is a modest risk factor (1.5–2 fold increased risk) for PDAC, NOD is a manifestation and harbinger of PDAC [34].

In our study, which included 69 patients with PDAC, diabetes was recognized in 36% cases. Among them, NOD was found in 14 (56%) with a mean overall duration of 6 months [43]. Because both DM and CP are risk factors for PDAC, the combination might particularly increase concern for progression to pancreatic cancer [66]. Meta-analyses have consistently shown a 1.5–2 times greater risk of PDAC in patients with long-standing (>5 years) diabetes [67,68]. In the meta-analysis of 20 epidemiologic studies, the pooled relative risk of PDAC for those whose diabetes was diagnosed at least 1 year prior to either diagnosis of pancreatic cancer or pancreatic cancer death was 2.1 (95% CI, 1.6–2.8) [69]. Many (but not all) cohort studies revealed that the risk of PDAC in the course of diabetes decreases with longer follow-ups (Table 1) [70,71,72,73,74].

The disturbed differentiation of islet cells was observed in animals with experimental PDAC but not CP and was suggested to be a precursor of PDAC. In a study by Schmied et al., in hamsters exposed to the pancreatic carcinogen N-nitrosobis(2-oxopropyl) amine, ductal cells, particularly those originating from periinsular and intrainsular regions, showed the highest sensitivity to this carcinogen; as the number of endocrine cells decreased, an increasing number of ductal cells appeared. Similar to cultured hamster islets, the formation of ductal cells occurred after seven days of culture, primarily in the center of the islets, where degeneration and lysis of endocrine cells begin. Therefore, it seems that necrosis acts as a stimulus for the formation of ductal cells. In these animals, the most PDACs develop within islets either from islet precursor stem cells or through the gradual differentiation of islet cells [75].

The correlation between the number of endocrine cells and the levels of hormones secreted at each time point clearly indicates that these hormones were produced and released by the cells in culture, rather than being secreted by dying cells. The neoplastic process began with the hyperplasia of intralobular ductular and interlobular ductal cells, accompanied by the formation of new islets. This was followed by an overproduction of both mature and immature islet cells along with their precursors at the islet periphery, as well as the emergence, distension, and proliferation of periinsular and intrainsular ductules. The hyperplasia, metaplasia, and malignant transformation of these periinsular, intrainsular ductules, and to a lesser extent, intercalated ductules, indicated their histogenetic relationship and their potential to regenerate embryonic tissue in response to carcinogenic stimuli. The resemblance of some induced lesions to changes seen in diabetes has been highlighted [76].

PP is a hormone predominantly secreted by the F cells in pancreatic islets and has been studied in patients with CP and PDAC [77]. Skrha determined the plasma concentrations of selected gastrointestinal hormones (GIP, GLP-1, PP, peptide YY (PYY), and neuropeptide Y (NPY), as well as cytokines, leptin, and adiponectin) in PDAC patients with and without diabetes and compared them with the levels found in type 2 diabetic patients without cancer. They examined the serum concentrations and found significantly lower serum concentrations of GIP in the PDAC patients with NOD or pre-diabetes (*n* = 76) or normal glycemic regulation (*n* = 18) compared with the patients with type 2 diabetes without PDAC and the control group (15.5 (3.7–64.5) or 6.5 (1.7–24.5) versus 39.8 (15.1–104.7) or 28.8 (7.4–112.2) ng/L, respectively; *p* < 0.001). The same trend was observed for PP (38.9 (10.2–147.9) or 28.1 (7.9–100.0) versus 89.1 (38.0–208.9) or 75.8 (30.1–190.6) ng/L, respectively; *p* < 0.01). The lowest levels of GIP and PP concentrations were found in the PDAC patients with NOD or pre-diabetes and weight loss > 2 kg (*p* < 0.001). The authors concluded that the serum concentrations of GIP and PP were lower in pancreatic cancer patients regardless of the degree of glucose intolerance compared with patients with type 2 diabetes and the healthy individuals in the control group [78]. In the case of NOD, especially when associated with weight loss, these changes may provide a new clue in the diagnosis of PDAC. However, whether PP directly induces lipolysis or differentially impacts subcutaneous and visceral adipose tissue compartments has not been investigated. This phenomenon is believed to be associated with the increased expression of proteases such as fibroblast activation protein α and dipeptidyl peptidase 4 in pancreatic cancer tissue [78,79].

## 5. The Role of Exosomes in PDAC

Exosomes are membrane vesicles of endocytic origin, and their size ranges from 30 to 150 nm. They are found in almost all body fluids, including blood, sweat, tears, urine, saliva, breast milk, ascites, and cerebrospinal fluid [80]. They transport various types of macromolecules from cells of different tissues and organs. The contents of exosomes are encapsulated within multivesicular body (MVB) membranes through inward invagination. Once MVBs are formed within the cells, some secreted MVBs fuse with lysosomes, leading to the degradation of MVBs and their contents. The secreted MVBs then merge with the plasma membrane, releasing exosomes into the extracellular environment [81]. The exosomes include proteins, nucleic acids, and lipids [82]. Exosomes are involved in antigen presentation, cell differentiation, and the growth, migration, and invasion of tumor cells. In cancers, exosomes may play a role in growth and metastasis by modulating the immune response, inhibiting the epithelial-mesenchymal transition, and facilitating angiogenesis [81]. Exosomes affect the functions of distant cell types and may alter the behavior of recipient cells by transferring bioactive molecules to them [28,83,84]. Released by a specific cell type, exosomes may operate within a defined microenvironment or even exert effects at a distance. The exchange of information between cells via exosomes is often bidirectional. Since exosomes carry molecules which are characteristic of their cell of origin, they can be utilized as biomarkers. Furthermore, because extracellular vesicles contain distinct patterns of mRNA, microRNA, long non-coding RNA, and occasionally genomic DNA, they have the potential to transfer genetic information which induces transient or persistent phenotypic changes in the recipient cells [84].

The pancreatic tumor microenvironment in humans is a complex that consists of immune cells, endothelial cells, and cancer-associated fibroblasts, that activate pancreatic stellate cells (PSCs). Recent insights have illuminated the considerable inter- and intra-tumoral heterogeneity exhibited by PSCs in the context of PDAC. The phenomenon of pancreatic diabetes is linked to the progressive loss and dysfunction of β cells, coupled with insulin resistance. Experimental investigations have indicated that exosomes derived from PDAC cells harbor factors that adversely affect β cell functionality and insulin sensitivity. Nevertheless, the precise role of stromal cells, particularly PSCs, in the etiology of pancreatic diabetes remains elusive. The exosomes from both cancer cells and PSCs may significantly influence β cell function and peripheral insulin resistance in humans. Cancer cells, along with the adjacent stromal cells, particularly PSCs, which are pivotal in generating the collagenous stroma characteristic of PDAC may release certain factors that operate through a ‘humoral’ mechanism. This could potentially suppress islet cell functionality from afar, away from the PanIN lesions or cancerous sites, and/or interfere with insulin signaling pathways, culminating in insulin resistance [31].

Wang et al. studied that exosomes derived from the KPC mouse PDAC cell line on insulin resistance within mouse skeletal myocytes, specifically C2C12 cells. Their findings revealed that KPC exosomes markedly impede glucose uptake in these myocytes. Through the application of miRNA microarray and Kyoto Encyclopedia of Genes and Genomes (KEGG) analysis, the researchers elucidated that these inhibitory effects were orchestrated by miR-450b-3p and miR-151-3p, which are encapsulated within the exosomes from cancer cells, and exert their influence via the PI3K/Akt/FoxO1 signaling cascade. Additionally, the study highlighted a pronounced downregulation of the glucose transporter GLUT4 in myocytes following exposure to KPC exosomes. Notably, the attenuation of FoxO1 expression was found to reverse the aforementioned effects induced by KPC exosomes, underscoring the pivotal role of this pathway in mediating insulin resistance [85].

It has been reported that of exosomes play a significant role in PDAC carcinogenesis. In a study by Melo et al., mass spectrometry analyses revealed the presence of a cell surface proteoglycan, glypican-1 (GPC1), which is notably abundant on exosomes derived from cancer cells. Glypican-1 is a membrane protein that plays a role in activating various growth factors. Researchers identified and isolated circulating exosomes (crExos) positive for GPC1 from the serum of cancer patients and mice using flow cytometry. These GPC1-positive crExos were specifically detected in the serum of individuals with pancreatic cancer, demonstrating high specificity and sensitivity for distinguishing healthy individuals, patients with benign pancreatic conditions, and those with PAnINs, early, and advanced stages of pancreatic cancer [86]. In a comparison between pancreatic cancer patients in stages I–IV, healthy individuals, and patients with benign pancreatic disease (BPD), the ROC curves showed that GPC1+ crExos acted as an almost perfect classifier, achieving an AUC of 1.0 (95% CI, 0.988–1.0). This classification resulted in a sensitivity of 100% (95% CI, 98.1–100%) and specificity of 100% (95% CI, 97.1–100%), with both positive and negative predictive values of 100% (95% CI, 98.1–100% and 95% CI, 86.8–100%, respectively). GPC1+ crExos demonstrated 100% sensitivity and specificity across all pancreatic cancer stages (in situ, stage I, and stages II–IV), highlighting their potential as biomarkers throughout the progression of pancreatic cancer and their application in early detection [86]. Additionally, measuring glypican levels can also predict survival, and this can be done using commercially available kits [87].

Exosomes play a significant role in material transport and signal transmission by releasing the specific components they carry into recipient cells. Exosomes derived from normal fibroblast-like mesenchymal cells were engineered to carry small interfering RNA (siRNA) or short hairpin RNA (shRNA) specific to oncogenic KRASG12D (iExosomes), a common mutation in pancreatic cancer. iExosome treatment inhibited cancer progression in various mouse models of pancreatic cancer and significantly improved the overall survival rates. The mutant form of the GTPase Kras is a crucial driver of pancreatic cancer but poses a challenging therapeutic target. It has been shown that the increased retention of exosomes in circulation, as opposed to liposomes, is attributed to CD47-mediated protection of exosomes from phagocytosis by monocytes and macrophages. In comparison with liposomes, iExosomes target oncogenic Kras with enhanced efficacy which relies on CD47 and is facilitated by micropinocytosis [88].

The potential roles of the tumor microenvironment (TME), the immune system, cancer-associated adipocytes, and PDAC-associated exosomes require further investigation [89]. A hypothesis was formulated that the cargo inducing lipolysis is transferred in exosomes released by PDAC and is responsible for paraneoplastic effects, such as diabetes. Therefore, this study investigated whether exosomes released by PDAC induce lipolysis in adipocytes and examined the role of ADM in PDAC exosomes as a mediator of this lipolysis. It has been shown that glucagon regulates exosome production in the endothelial cells of adipose tissues. Moreover, exosomes cause hyperglycemia because of their impact on β cells as well as lipolysis in the subcutaneous fat tissue and further weight loss in pancreatic cancer patients. PDAC is also linked to the release of abnormal exosomes that may carry factors such as ADM, which induces β cell dysfunction and activates MAPK and p38-MAPK in adipocytes, resulting in increased lipolysis in these cells [27,35]. In a study of Javeed et al., the subcutaneous adipose tissue from non-cancer individuals after surgeries was obtained. Normal pancreatic tissue samples were collected from autopsy specimens. Serum samples were isolated for exosome isolation, and a pancreatic resection sample was obtained to establish a cell line and for immunohistochemical studies. The study compared exosomes from PDAC patients, which activated lipolysis in human subcutaneous adipocytes, to exosomes from non-PDAC control individuals, as demonstrated in a glycerol test in the supernatants. The levels of ADM were significantly elevated (1.7 ± 0.29 times) in the exosomes from PDAC patients compared with those from non-PDAC control individuals. Additionally, the average number of exosomes in PDAC patients was 11 billion per ml of serum, with an average excess of 8 µg of protein in the exosomal fraction per ml of serum. The authors confirmed the role of ADM in tumor and pancreatic diabetes-related lipolysis due to its potential to induce β cell dysfunction and its association with pancreatic tumor-induced diabetes. ADM was found to have varying effects on lipolysis, depending on the cell context. When considering mouse and human adipocytes, it was observed that both recombinant and exosomal ADM promote lipolysis by activating p38 and p44/42. This effect was abolished by ADM inhibitor treatment [27]. Sagar et al. were the first to demonstrate that pancreatic cancer cells (PCCs) lines preferentially release exosomes rather than other forms of extracellular vesicles, and they were present in both the portal and peripheral venous blood of PDAC patients. Additionally, it was determined that exosomes derived from PANC-1 PCC cell lines can be internalized by β cells within 48 h of coincubation. Exosomes were identified as the predominant extracellular vesicles secreted by PDAC into culture media and human plasma [35].

Liquid chromatography multiple reaction monitoring mass spectrometry (LC-MRM-MS)-based targeted quantitative lipidomics methods were utilized to identify potential biomarkers in serum exosomes from pancreatic cancer patients. The analysis revealed that the small molecule metabolites present in exosomes were linked to carcinogenesis. The findings indicated an altered lipid profile in those patients’ exosomes, suggesting their potential use as diagnostic biomarkers for PDAC and providing insights into the underlying pathological mechanisms of this cancer. In a study by Tao et al., which examined 270 lipids across 20 lipid species, significant dysregulation was observed in the serum exosome expression of PDAC patients compared with healthy controls. The results highlighted associations of lysophosphatidylcholine (LysoPC), phosphatidylcholine (PC), and phosphatidylethanolamine (PE) with the tumor stage, CA19-9, CA242, and tumor size. Additionally, PE was significantly correlated with overall patient survival. LysoPCs, which constitute a small fraction of phospholipids in cell membranes (≤3%) and blood plasma (8–12%), are rapidly metabolized by lysophospholipase and LPC-acyltransferase, resulting in their short-lived presence in vivo [90].

PDAC is linked with the production of exosomes rather than other extracellular vesicles. This raises the prospect that exosomes from PDAC might play a role in insulin resistance, β cell dysfunction, and fat breakdown, potentially accounting for the increased prevalence of NOD in PDAC. Exosomes derived from pancreatic cancer cells induce insulin resistance in skeletal muscles, as extrapolated from observations that such exosomes inhibit insulin-PI3K-Akt signaling in C2C12 myotubes, thereby disrupting GLUT4 uptake [91]. NOD frequently occurs before symptoms of cachexia, indicating that cachexia does not contribute to NOD. Notably, neoplasm resection retrieve insulin sensitivity, suggesting that exosomes from PDAC may play a role in insulin resistance, β cell failure, and lipolysis, which could explain the high prevalence of NOD in PDAC. Therefore, NOD can be partially regarded as an exosome-related condition associated with PDAC. NOD can therefore be considered, in part, a PDAC-associated exosomopathy. Diabetes in the course of pancreatic cancer triggers both insulin resistance and dysfunction of β cells, ultimately leading to loss of glycemic control, which serves as a precursor to the development of PDAC [92]. Further examinations should clarify if exosomes from PDAC also trigger insulin resistance, a mechanism involved in pancreatic cancer-related diabetes, and whether the loss of β cell mass in CP is a result of exosomes targeting the endocrine islets. It is also important to investigate whether exosome-delivered cargo proteins, apart from ADM, or the RNAs and microRNAs contained within the exosomes contribute to these or other aspects of PDAC pathobiology. Additionally, it is essential to determine if ADM within the tumor microenvironment can harm β cells and whether the activation of ER stress by exosome-delivered ADM is linked to classical upregulation of protein kinase R (PKR)-like endoplasmic reticulum kinase (PERK) [93].

The work described in the article contains data obtained from a relatively small number of patients. Thus, it can be used to systematize which potential microRNAs are repeated, and these molecules can be typed as biomarkers, which should then be tested on an adequately larger and diversified dataset in terms of both sex, race, age, and other factors, as well as other types of cancer. It is also possible to think about performing a meta-analysis using the sequencing and microarray data collected in databases made available by other researchers and based on this work, followed by selecting the molecules with the highest expression in samples with the described cancer. If the expression differs significantly between populations of people living in different regions of the world or people of a different race, then appropriate diagnostic panels should be prepared for each of the groups. In addition to diagnostic functions, exosomes can also be used to treat pancreatic cancer. Exosomes are one of the ways of transporting various biological materials in the body, and this is why, when using them to deliver drugs, we would expect the smallest possible perturbations at the cellular level. However, if we are talking about microRNA, then if we have a panel of molecules which participate in the activation of anti-cancer or anti-inflammatory pathways, then we can administer more of them to a given place as part of the treatment and promote the desired pathways more than others.

## 6. Conclusions

Despite the rapid progress in research, pancreatic cancer remains a challenge in terms of its early diagnosis. Differentiating type 2 diabetes from the one which occurs in the early stage of cancer may allow for recognition of neoplastic lesions and improve the prognoses among these patients. Despite the research, a marker of diabetes accompanying cancer is still being sought. Many authors of multi-center studies are in the process of searching for this, and our results also lead in the direction of finding it. Further studies on exosomes biology in PDAC brings hope for their potential application in the diagnosis and treatment of this neoplasm. PDAC exosomes may be novel early diagnostic markers for PDAC. As previously mentioned, there is an urgent need for the development of noninvasive, pre-diagnostic validated biomarkers for the longitudinal surveillance of high-risk individuals, as well as imaging modalities capable of identifying pancreatic cancer at an early stage. Determining the carefully selected groups of patients for PDAC screening may be helpful for early detection of pancreatic cancer and application of the optimal treatment regimen, resulting in improved patient survival.

## Figures and Tables

**Table 1:** Incidence of different endocrine function pathologies in the course of PDAC.

Type of Endocrine Disorder	Incidence in PDAC	References
Longstanding diabetes	4–20%	[15,62,63,64,65,66,67]
Pancreatic cancer-associated diabetes	28–47%	[11,13,41]
NOD	56–85%	[11,12,13,14,22,23,24,25,36]
IGT	17–38%	[11,40,41]
Hyperglycemia	17–85%	[11,12,13,14,41,42,43]

Pancreatic ductal adenocarcinoma (PDAC), new-onset diabetes (NOD), and impaired glucose tolerance (IGT).

## Data Availability

The data presented in this study are available on request from the corresponding author.
